# Supplementary material for: Unidirectional versus bidirectional brushing: Simulating wind influence on Arabidopsis thaliana
Source: Quant Plant Biol. 2022 Apr 11;3:e7. doi: 10.1017/qpb.2021.14 (PMC10095948; doi:10.1017/qpb.2021.14)
Supplement: Supplementary file 1 [file qpbsup.zip › S263288282100014Xsup001.docx]

**Supplementary material 1**

Plant morphology raw data from unidirectional and bidirectional brushing experiments presented as bar plots in Figure 1:

L – length of the primary inflorescence stem

Nst – total number of stems (basal branches)

Nbr – total number of branches (excluding basal branches)

**Unidirectional brushing**

|  | **Control group** | | | **Experimental group 1 (brushed with textured jute fabric)** | | | **Experimental group 2 (brushed with smooth plastic)** | | |
| --- | --- | --- | --- | --- | --- | --- | --- | --- | --- |
| **plant** | **L, mm** | **Nst** | **Nbr** | **L, mm** | **Nst** | **Nbr** | **L, mm** | **Nst** | **Nbr** |
| 01 | 378 | 8 | 35 | 267 | 7 | 31 | 314 | 7 | 36 |
| 02 | 381 | 8 | 31 | 275 | 8 | 33 | 257 | 7 | 31 |
| 03 | 399 | 7 | 33 | 267 | 7 | 30 | 289 | 7 | 35 |
| 04 | 348 | 7 | 32 | 317 | 7 | 32 | 281 | 7 | 33 |
| 05 | 344 | 7 | 37 | 321 | 8 | 35 | 260 | 8 | 37 |
| 06 | 356 | 8 | 34 | 324 | 8 | 37 | 315 | 7 | 36 |
| 07 | 359 | 8 | 33 | 271 | 7 | 35 | 302 | 7 | 32 |
| 08 | 381 | 7 | 30 | 280 | 7 | 36 | 281 | 7 | 33 |
| 09 | 401 | 6 | 29 | 304 | 7 | 28 | 310 | 7 | 29 |
| 10 | 301 | 7 | 36 | 299 | 8 | 34 | 299 | 8 | 34 |
| 11 | 401 | 7 | 31 | 282 | 7 | 35 | 315 | 7 | 31 |
| 12 | 398 | 6 | 34 | 322 | 6 | 30 | 319 | 7 | 33 |
| 13 | 399 | 7 | 32 | 290 | 7 | 31 | 297 | 8 | 32 |
| 14 | 405 | 6 | 36 | 321 | 7 | 32 | 335 | 8 | 37 |
| 15 | 372 | 7 | 37 | 346 | 7 | 34 | 328 | 7 | 34 |
| 16 | 390 | 8 | 40 | 354 | 8 | 37 | 305 | 8 | 33 |
| 17 | 319 | 6 | 29 | 282 | 7 | 34 | 290 | 6 | 29 |
| 18 | 361 | 7 | 35 | 351 | 8 | 34 | 337 | 8 | 39 |
| 19 | 356 | 8 | 33 | 316 | 8 | 35 | 261 | 7 | 36 |
| 20 | 320 | 8 | 39 | 251 | 7 | 29 | 332 | 7 | 31 |

**Bidirectional brushing**

|  | **Control group** | | | **Experimental group 1 (brushed with textured jute fabric)** | | | **Experimental group 2 (brushed with smooth plastic)** | | |
| --- | --- | --- | --- | --- | --- | --- | --- | --- | --- |
| **plant** | **L, mm** | **Nst** | **Nbr** | **L, mm** | **Nst** | **Nbr** | **L, mm** | **Nst** | **Nbr** |
| 01 | 364 | 8 | 33 | 269 | 7 | 33 | 295 | 6 | 28 |
| 02 | 344 | 7 | 38 | 274 | 7 | 32 | 260 | 7 | 30 |
| 03 | 350 | 7 | 35 | 254 | 7 | 28 | 309 | 8 | 34 |
| 04 | 365 | 8 | 36 | 270 | 7 | 32 | 250 | 6 | 31 |
| 05 | 374 | 7 | 31 | 256 | 6 | 31 | 263 | 6 | 33 |
| 06 | 420 | 8 | 41 | 266 | 8 | 39 | 244 | 7 | 32 |
| 07 | 401 | 7 | 35 | 285 | 9 | 44 | 272 | 6 | 31 |
| 08 | 344 | 7 | 28 | 254 | 6 | 30 | 266 | 7 | 31 |
| 09 | 402 | 7 | 39 | 280 | 7 | 36 | 280 | 6 | 36 |
| 10 | 354 | 7 | 31 | 256 | 6 | 29 | 269 | 6 | 32 |
| 11 | 367 | 6 | 32 | 269 | 8 | 32 | 264 | 8 | 29 |
| 12 | 358 | 8 | 33 | 253 | 7 | 28 | 270 | 7 | 32 |
| 13 | 330 | 7 | 30 | 265 | 7 | 34 | 264 | 6 | 34 |
| 14 | 339 | 7 | 37 | 270 | 7 | 39 | 278 | 8 | 29 |
| 15 | 366 | 7 | 23 | 268 | 6 | 26 | 269 | 8 | 36 |
| 16 | 338 | 8 | 30 | 274 | 7 | 32 | 273 | 7 | 30 |
| 17 | 329 | 7 | 29 | 270 | 8 | 32 | 266 | 7 | 35 |
| 18 | 333 | 7 | 30 | 272 | 7 | 28 | 262 | 7 | 32 |
| 19 | 321 | 8 | 34 | 241 | 7 | 31 | 261 | 7 | 30 |
| 20 | 316 | 6 | 30 | 252 | 6 | 28 | 243 | 6 | 29 |
