# Supplementary material for: Unidirectional versus bidirectional brushing: Simulating wind influence on Arabidopsis thaliana
Source: Quant Plant Biol. 2022 Apr 11;3:e7. doi: 10.1017/qpb.2021.14 (PMC10095948; doi:10.1017/qpb.2021.14)
Supplement: Supplementary file 1 [file qpbsup.zip › S263288282100014Xsup002.docx]

**Supplementary material 2**

Raw data for the modulus of elasticity (*E*) of both tested parts of the Arabidopsis primary inflorescence stem from unidirectional and bidirectional brushing experiments presented as bar plots in Figure 2.

E_bot_ – modulus of elasticity determined for the bottom part of the primary inflorescence stem

E_top_– modulus of elasticity determined for the top part of the primary inflorescence stem

**Unidirectional brushing**

|  | **Control group** | | **Experimental group 1 (brushed with textured jute fabric)** | | **Experimental group 2 (brushed with smooth plastic)** | |
| --- | --- | --- | --- | --- | --- | --- |
| **plant** | **E_bot_, MPa** | **E_top_, MPa** | **E_bot_, MPa** | **E_top_, MPa** | **E_bot_, MPa** | **E_top_, MPa** |
| 01 | 1063.0 | 503.65 | 683.40 | 465.93 | 806.87 | 497.72 |
| 02 | 942.07 | 464.44 | 1170.1 | 348.72 | 928.25 | 530.72 |
| 03 | 1228.7 | 593.26 | 973.56 | 353.45 | 694.38 | 483.02 |
| 04 | 991.38 | 602.77 | 867.81 | 430.47 | 552.59 | 290.68 |
| 05 | 1125.2 | 488.58 | 764.03 | 383.65 | 822.74 | 566.86 |
| 06 | 1146.2 | 667.04 | 861.83 | 490.82 | 941.89 | 568.51 |
| 07 | 1316.0 | 674.08 | 842.82 | 559.31 | 982.77 | 678.29 |
| 08 | 1153.7 | 700.84 | 830.14 | 741.18 | 1071.0 | 746.58 |
| 09 | 1376.4 | 639.03 | 858.38 | 806.71 | 968.82 | 562.31 |
| 10 | 831.77 | 314.92 | 892.96 | 744.95 | 1143.9 | 519.23 |

**Bidirectional brushing**

|  | **Control group** | | **Experimental group 1 (brushed with textured jute fabric)** | | **Experimental group 2 (brushed with smooth plastic)** | |
| --- | --- | --- | --- | --- | --- | --- |
| **plant** | **E_bot_, MPa** | **E_top_, MPa** | **E_bot_, MPa** | **E_top_, MPa** | **E_bot_, MPa** | **E_top_, MPa** |
| 01 | 1279.6 | 492.62 | 753.13 | 551.67 | 521.70 | 518.38 |
| 02 | 1008.5 | 583.94 | 1003.8 | 727.07 | 760.27 | 618.21 |
| 03 | 1081.2 | 728.00 | 605.70 | 511.21 | 727.85 | 628.44 |
| 04 | 1334.9 | 785.37 | 755.76 | 363.24 | 600.69 | 552.82 |
| 05 | 1026.3 | 611.86 | 641.95 | 615.68 | 658.24 | 520.43 |
| 06 | 939.05 | 412.89 | 870.92 | 470.02 | 740.87 | 506.72 |
| 07 | 846.68 | 523.54 | 534.27 | 349.55 | 772.11 | 464.64 |
| 08 | 1366.7 | 476.79 | 519.06 | 652.28 | 922.29 | 706.86 |
| 09 | 941.42 | 601.25 | 506.03 | 505.29 | 479.00 | 505.27 |
| 10 | 1267.3 | 390.98 | 828.82 | 587.86 | 726.78 | 514.30 |
