# Supplementary material for: Unidirectional versus bidirectional brushing: Simulating wind influence on Arabidopsis thaliana
Source: Quant Plant Biol. 2022 Apr 11;3:e7. doi: 10.1017/qpb.2021.14 (PMC10095948; doi:10.1017/qpb.2021.14)
Supplement: Supplementary file 1 [file qpbsup.zip › S263288282100014Xsup003.docx]

**Supplementary material 3**

Supplementary Figure S3-1: The effect of mechanical perturbations by different types of brushing treatment and brushing material on Arabidopsis morphology. (A) Primary inflorescence stem length. (B) Number of stems. (C) Number of branches. The legend in part (A) applies to all parts of this figure. Experimental group 1 was brushed with textured jute fabric while experimental group 2 with a smooth plastic in both experiments. Statistically significant differences ($P\leq0.05$) between groups were identified using ANOVA with a post hoc Tukey test. * marks statistically significant difference compared with the control group in the respective experiment. The raw data for both experiments is presented below.

Plant morphology raw data from additional unidirectional and bidirectional brushing experiments presented as bar plots in Supplementary Figure S3-1:

L – length of the primary inflorescence stem

Nst – total number of stems (basal branches)

Nbr – total number of branches (excluding basal branches)

**Unidirectional brushing**

|  | **Control group** | | | **Experimental group 1 (brushed with textured jute fabric)** | | | **Experimental group 2 (brushed with smooth plastic)** | | |
| --- | --- | --- | --- | --- | --- | --- | --- | --- | --- |
| **plant** | **L, mm** | **Nst** | **Nbr** | **L, mm** | **Nst** | **Nbr** | **L, mm** | **Nst** | **Nbr** |
| 01 | 259 | 5 | 15 | 245 | 4 | 20 | 274 | 6 | 33 |
| 02 | 300 | 6 | 16 | 234 | 4 | 13 | 256 | 4 | 11 |
| 03 | 308 | 6 | 45 | 243 | 5 | 31 | 254 | 5 | 25 |
| 04 | 278 | 4 | 6 | 239 | 5 | 20 | 251 | 5 | 17 |
| 05 | 290 | 5 | 18 | 232 | 5 | 15 | 230 | 5 | 19 |
| 06 | 302 | 6 | 21 | 220 | 4 | 15 | 209 | 5 | 15 |
| 07 | 270 | 6 | 18 | 231 | 5 | 19 | 246 | 4 | 21 |
| 08 | 226 | 5 | 11 | 225 | 5 | 18 | 225 | 4 | 11 |
| 09 | 307 | 5 | 20 | 228 | 5 | 12 | 203 | 5 | 26 |
| 10 | 294 | 7 | 14 | 246 | 5 | 15 | 229 | 5 | 16 |
| 11 | 241 | 6 | 18 | 208 | 6 | 12 | 230 | 4 | 16 |
| 12 | 270 | 5 | 18 | 241 | 5 | 16 | 247 | 5 | 12 |
| 13 | 260 | 6 | 14 | 237 | 5 | 18 | 224 | 5 | 13 |
| 14 | 302 | 5 | 26 | 246 | 5 | 6 | 208 | 4 | 18 |
| 15 | 228 | 3 | 11 | 219 | 6 | 19 | 190 | 4 | 17 |
| 16 | 259 | 4 | 23 | 194 | 5 | 16 | 186 | 5 | 13 |
| 17 | 253 | 4 | 16 | 189 | 4 | 17 | 211 | 5 | 5 |
| 18 | 247 | 3 | 13 | 213 | 5 | 16 | 190 | 5 | 13 |
| 19 | 231 | 4 | 21 | 200 | 4 | 8 | 175 | 4 | 6 |
| 20 | 241 | 5 | 19 | 214 | 4 | 14 | 221 | 5 | 22 |

**Bidirectional brushing**

|  | **Control group** | | | **Experimental group 1 (brushed with textured jute fabric)** | | | **Experimental group 2 (brushed with smooth plastic)** | | |
| --- | --- | --- | --- | --- | --- | --- | --- | --- | --- |
| **plant** | **L, mm** | **Nst** | **Nbr** | **L, mm** | **Nst** | **Nbr** | **L, mm** | **Nst** | **Nbr** |
| 01 | 350 | 5 | 28 | 269 | 7 | 33 | 285 | 6 | 30 |
| 02 | 328 | 6 | 27 | 270 | 5 | 29 | 224 | 5 | 32 |
| 03 | 337 | 6 | 25 | 252 | 6 | 42 | 271 | 5 | 41 |
| 04 | 410 | 7 | 34 | 259 | 8 | 34 | 253 | 6 | 30 |
| 05 | 346 | 6 | 35 | 270 | 6 | 26 | 290 | 7 | 31 |
| 06 | 341 | 6 | 39 | 246 | 6 | 32 | 245 | 6 | 25 |
| 07 | 356 | 4 | 30 | 232 | 7 | 30 | 271 | 6 | 27 |
| 08 | 327 | 6 | 39 | 225 | 6 | 28 | 236 | 6 | 24 |
| 09 | 343 | 6 | 28 | 241 | 6 | 32 | 230 | 6 | 29 |
| 10 | 358 | 6 | 33 | 260 | 5 | 33 | 286 | 6 | 26 |
| 11 | 320 | 6 | 26 | 280 | 6 | 23 | 260 | 6 | 33 |
| 12 | 369 | 7 | 30 | 282 | 7 | 27 | 230 | 5 | 28 |
| 13 | 383 | 6 | 39 | 252 | 6 | 31 | 231 | 6 | 31 |
| 14 | 351 | 8 | 35 | 272 | 6 | 28 | 241 | 6 | 28 |
| 15 | 316 | 6 | 22 | 257 | 5 | 22 | 237 | 7 | 34 |
| 16 | 386 | 6 | 42 | 226 | 7 | 36 | 282 | 6 | 29 |
| 17 | 320 | 7 | 31 | 235 | 6 | 32 | 250 | 7 | 39 |
| 18 | 362 | 5 | 21 | 253 | 6 | 33 | 242 | 7 | 28 |
| 19 | 321 | 9 | 30 | 251 | 7 | 25 | 240 | 7 | 32 |
| 20 | 309 | 7 | 34 | 216 | 6 | 28 | 254 | 6 | 29 |

Supplementary Table S3-1: The effect of mechanical perturbations by different types of brushing and brushing material on Arabidopsis morphology.

|  | Unidirectional brushing | | | Bidirectional brushing | | |
| --- | --- | --- | --- | --- | --- | --- |
|  | control | experimental group 1 | experimental group 2 | control | experimental group 1 | experimental group 2 |
|  | whole plant | | | | | |
| fresh biomass, g | 6.04±0.95 | 5.33±0.35 | 5.74±0.49 | 7.99±1.17 | 7.22±0.97 | 6.95±1.27 |
| dry biomass, g | 0.55±0.07 | 0.50±0.05 | 0.52±0.06 | 0.85±0.21 | 0.71±0.11 | 0.66±0.15 |
|  | bottom part of the stem | | | | | |
| D, mm | 1.20±0.15 | 1.04±0.10* | 1.10±0.09 | 1.86±0.25 | 1.68±0.26 | 1.71±0.22 |
| EI, N mm^2^ | 117±70 | 49±18* | 60±17* | 625±281 | 268±170* | 328±219* |
|  | top part of the stem | | | | | |
| D, mm | 1.01±0.08 | 0.99±0.10 | 1.06±0.07 | 1.07±0.06 | 1.09±0.06 | 1.11±0.08 |
| EI, N mm^2^ | 22±6 | 21±6 | 27±8 | 31±10 | 30±8 | 32±8 |

Experimental group 1 was brushed with textured jute fabric while experimental group 2 with smooth plastic in both experiments. Values are presented as mean±SD with n=10 for each case. Statistically significant differences ($P\leq0.05$) between groups were identified using ANOVA with a post hoc Tukey test.

* marks statistically significant difference compared with the control group in the respective experiment.

Supplementary Figure S3-2: The effect of mechanical perturbations by different types of brushing treatment and brushing material on the intensive mechanical properties of Arabidopsis primary inflorescence stem. (A) Modulus of elasticity of the bottom part of the stem. (B) Modulus of elasticity of the top part of the stem. The legend in part (B) applies to both parts of this figure. Experimental group 1 was brushed with textured jute fabric while experimental group 2 with smooth plastic in both experiments. Statistically significant differences ($P\leq0.05$) between groups were identified using ANOVA with a post hoc Tukey test. * marks statistically significant difference compared with the control group in the respective experiment. The raw data for both experiments is presented below.

Raw data for the modulus of elasticity (*E*) of both tested parts of the Arabidopsis primary inflorescence stem presented as bar plots in Supplementary Figure S3-2.

E_bot_ – modulus of elasticity determined for the bottom part of the primary inflorescence stem

E_top_– modulus of elasticity determined for the top part of the primary inflorescence stem

**Unidirectional brushing**

|  | **Control group** | | **Experimental group 1 (brushed with textured jute fabric)** | | **Experimental group 2 (brushed with smooth plastic)** | |
| --- | --- | --- | --- | --- | --- | --- |
| **plant** | **E_bot_, MPa** | **E_top_, MPa** | **E_bot_, MPa** | **E_top_, MPa** | **E_bot_, MPa** | **E_top_, MPa** |
| 01 | 807.76 | 268.63 | 914.55 | 695.73 | 794.80 | 315.48 |
| 02 | 881.32 | 702.85 | 913.99 | 609.71 | 927.57 | 284.07 |
| 03 | 1240.0 | 337.52 | 883.44 | 520.85 | 827.29 | 707.86 |
| 04 | 1001.6 | 425.89 | 808.43 | 423.34 | 675.62 | 438.25 |
| 05 | 1048.3 | 795.74 | 718.07 | 319.02 | 938.89 | 283.94 |
| 06 | 909.48 | 366.68 | 808.75 | 543.81 | 808.44 | 496.19 |
| 07 | 889.07 | 332.09 | 903.26 | 363.14 | 765.75 | 505.47 |
| 08 | 1021.8 | 319.60 | 706.43 | 447.18 | 908.98 | 499.03 |
| 09 | 1191.0 | 453.76 | 671.89 | 330.35 | 721.85 | 452.44 |
| 10 | 1133.7 | 368.26 | 855.65 | 259.28 | 689.18 | 316.73 |

**Bidirectional brushing**

|  | **Control group** | | **Experimental group 1 (brushed with textured jute fabric)** | | **Experimental group 2 (brushed with smooth plastic)** | |
| --- | --- | --- | --- | --- | --- | --- |
| **plant** | **E_bot_, MPa** | **E_top_, MPa** | **E_bot_, MPa** | **E_top_, MPa** | **E_bot_, MPa** | **E_top_, MPa** |
| 01 | 1130.3 | 540.15 | 616.06 | 572.33 | 709.06 | 398.36 |
| 02 | 1017.9 | 562.73 | 896.07 | 715.97 | 669.37 | 369.47 |
| 03 | 762.49 | 349.74 | 497.24 | 408.30 | 488.49 | 416.65 |
| 04 | 984.17 | 700.19 | 616.60 | 578.99 | 377.33 | 281.30 |
| 05 | 1203.8 | 506.79 | 700.92 | 406.18 | 706.34 | 369.91 |
| 06 | 1187.1 | 686.92 | 542.25 | 342.50 | 548.73 | 504.25 |
| 07 | 909.19 | 642.96 | 535.87 | 321.75 | 654.55 | 558.20 |
| 08 | 934.38 | 273.03 | 631.47 | 505.76 | 1004.2 | 718.70 |
| 09 | 904.80 | 488.36 | 730.95 | 339.58 | 611.59 | 338.59 |
| 10 | 840.32 | 250.84 | 321.25 | 296.31 | 831.67 | 545.30 |

Supplementary Table S3-2: The effect of mechanical perturbations by different types of brushing and brushing mate- rial on the contribution of anatomical tissues to the total cross-sectional area of the primary inflorescence stem of Arabidopsis.

|  | Unidirectional brushing | | | Bidirectional brushing | | |
| --- | --- | --- | --- | --- | --- | --- |
|  | control | experimental group 1 | experimental group 2 | control | experimental group 1 | experimental group 2 |
|  | bottom part of the stem | | | | | |
| Pith | 37.06±4.01 | 36.66±3.24 | 37.54±2.12 | 44.78±1.84 | 47.78±1.09 | 46.61±2.94 |
| Lignified tissues | 28.52±4.19 | 23.91±2.20* | 24.02±2.08* | 28.44±1.88 | 22.04±2.70* | 23.36±2.69* |
| Cortex & epidermis | 34.42±4.20 | 39.43±3.45* | 38.44±3.48* | 26.78±2.23 | 30.18±2.64* | 30.03±2.34* |
|  | top part of the stem | | | | | |
| Pith | 44.61±3.32 | 42.39±3.04 | 42.93±3.42 | 44.56±2.77 | 44.18±2.16 | 43.81±2.08 |
| Lignified tissues | 22.42±1.84 | 22.79±1.37 | 21.68±1.84 | 23.42±1.84 | 22.26±1.63 | 22.17±2.49 |
| Cortex & epidermis | 32.97±3.10 | 34.82±2.67 | 35.39±3.09 | 32.02±2.12 | 33.56±1.51 | 34.02±2.67 |

Experimental group 1 was brushed with textured jute fabric while experimental group 2 with smooth plastic in both experiments. Values are presented as mean±SD with n=10 for each case. Statistically significant differences ($P\leq0.05$) between groups were identified using ANOVA with a post hoc Tukey test.

* marks statistically significant difference compared with the control group in the respective experiment.

Supplementary Figure S3-3: (A) Arabidopsis does not exhibit any tropic response in the bidirectional brushing experiment. (B) Arabidopsis exhibits a positive tropic response in the unidirectional brushing experiment.
